# Call for Action: High Rates of Depression in the Pediatric Celiac Disease Population Impacts Quality of Life

**DOI:** 10.1097/PG9.0000000000000074

**Published:** 2021-07-22

**Authors:** Hilary Jericho, Narmeen Khan, Jonathan Cordova, Naire Sansotta, Stefano Guandalini, Kate Keenan

**Affiliations:** From the * Department of Pediatrics, Division of Gastroenterology, Hepatology and Nutrition, Celiac Disease Center, University of Chicago Medicine, Comer Children’s Hospital, Chicago, IL; † Department of Pediatrics, NorthShore University HealthSystem, Evanston, IL; ‡ Paediatric Hepatology, Gastroenterology and Transplantation, Hospital Papa Giovanni XXIII, Bergamo, Italy; § Department of Psychiatry and Behavioral Neuroscience, University of Chicago Medicine, Chicago, IL.

**Keywords:** adolescents, celiac disease, children, depression, quality of life

## Abstract

To test the impact of celiac disease (CD) and depression symptoms on quality of life in adolescent patients.

**Methods:**

We conducted a prospective survey of 12- to 18-year-old celiac patients and their caregivers between January 2015 and November 2016. Enrolled parents and youth completed standard measures of adjustment to celiac disease, depression, and quality of life.

**Results:**

We enrolled 105 patients with CD and their parents. Both parents and youth reported high levels of depression symptoms. There were no associations between age, duration of CD, or following a gluten-free diet (GFD) and quality of life. No significant associations were found between adolescent perception of CD state and quality of life; parental report of adolescent’s adjustment to CD; and youth report of quality of life were modestly associated (r = 0.19, *P* ≤ 0.05). Moderate associations were observed between adolescent reports of depression and quality of life (r = 0.59, *P* < 0.01) and between parental reports of adolescent depression and quality of life (r = 0.41, *P* = 0.01). Only depressive symptoms by youth and parent report, however, and not adjustment to celiac, explained unique variance in quality of life.

**Conclusion:**

Adolescents with CD report levels of depression comparable to those reported by adolescents seeking mental health services. Length of time living with CD, or on GFD, age at diagnosis and perception of disease state do not appear to contribute to depression. High rates of depression may impact CD prognosis, therefore, screening for depression in adolescents with CD appears critical. Identification and intervention of depression may lead to improved adherence to the GFD during emerging adulthood.

**What Is Known**

- A lifelong gluten-free diet (GFD) is the only effective treatment for celiac disease (CD).
- Adults with CD report reduced quality of life on a gluten-free diet as compared to healthy controls.

**What Is New**

- Age, duration with celiac disease, or duration on a gluten-free diet are not associated with adolescent celiac patients’ reports of quality of life.
- Depression scores in celiac patients are on average high and comparable to those reported from psychiatric clinic samples of adolescents diagnosed with mental health disorders.
- Depression is the only statistically significantly correlate of quality of life in adolescent celiac patients.

## INTRODUCTION

Celiac disease (CD) is a complex autoimmune disease triggered by the ingestion of gluten (the major storage protein in wheat, barley, and rye) in genetically predisposed individuals, causing elevated titers of celiac-specific autoantibodies and resulting in variable degrees of small intestine inflammation and a wide range of gastrointestinal and extraintestinal manifestations (1).

In studies of adults, reduced quality of life has been reported among patients with CD on a gluten-free diet (GFD). Moreover, depression, anxiety, and poor coping strategies, as opposed to gastrointestinal symptoms, account for not only a lower quality of life (2), but also to worse rates of compliance to the GFD, which is necessary for optimal control of the disease (3) and reduction of long-term harmful sequelae.

We aimed to examine psychosocial functioning among adolescents with celiac disease, a particularly vulnerable population to noncompliance with the behavioral regimen required of celiac patients, to better understand which factors contribute to a lower quality of life and potentially to poor adherence to a gluten-free diet (4).

## METHODS

### Patients and Data Collection

We recruited 101 adolescent celiac patients, ages 12–18 years (42 males), and their caregivers at the University of Chicago Celiac Center between January 2015 and November 2016. All participants had a confirmed diagnosis of celiac disease according to the present guidelines (1). The criteria for inclusion in our study were as follows: positive serology and Marsh 1–3 findings on biopsy; tissue transglutaminase IgA more than 10 times normal with positive endomysial antibody with or without a biopsy; or positive serology and skin biopsy for dermatitis herpetiformis (DH). In the case of IgA deficiency, deamidated gliadin peptide (DGP) IgG was used. Age at celiac diagnosis, age at survey, sex, years on the GFD, and serologies at the time of the survey were recorded. Enrolled parents and youth completed the Child Symptom Inventory-4 (CSI-4) to assess for depression, the Children’s Symptom Inventory to assess depression symptoms (5), and the Celiac Disease Quality of Life (CDDUX), a 12-item questionnaire that elicits information about those aspects of life that are influenced by CD including 6 items on diet (e.g., “having to adhere to a life-long diet,” “not being able to eat anything I want”) (6). Youth also completed the Pediatric Quality of Life Survey-30 (7), with higher scores indicating lower quality of life.

### Statistical Methods

Spearman’s rho was used to test bivariate associations, and linear regressions were computed to test unique contribution of hypothesized variables on quality of life.

## RESULTS

Descriptive statistics are provided in Table 1. The average age at diagnosis was 10.6 years (standard deviation = 1.8 years). The average age at the time of the survey was 14.5 years (standard deviation = 3.7 years). The mean duration on the GFD at the time of the survey was 3.9 years (standard deviation = 3.7 years). Thirty-one patients reported gastrointestinal symptoms at the time of the survey. Twenty-four patients (24%) had ongoing elevation of their TTG IgA at the time of the survey, ranging from 1.5 to 9 times the upper level of normal. In all patients, the level at the time of the survey was improved from the level at the time of diagnosis with the exception of 2 patients for which the survey was completed at the time of diagnosis, suggestive of adherence to the GFD.

**TABLE 1.** Descriptive statistics (N = 101)

	N	%
Male sex	42	42.0
	Mean	SD
Age at time of survey	14.46	1.81
Age at diagnosis	10.62	3.69
Years on GFD	3.89	3.74
Depression score (youth)	13.14	3.40
Depression score (parent)	12.16	3.20
Adjustment to celiac (youth)	29.19	6.67
Adjustment to celiac (parent)	27.92	6.53
Peds quality of life (youth)	41.03	13.28

GFD = gluten-free diet.

Youth and their parents reported relatively high depression scores (means = 13.1 and 12.2, respectively, see Table 1). These scores are comparable to those reported from a psychiatric clinic sample of adolescents with diagnosed mental health disorders (means = 16.2 and 13.3, respectively) (8) and twice as high as scores reported in a community sample of White and Black American girls (means = 6.6 and 6.9, respectively) (9). The most commonly endorsed symptoms by youth report were sad mood and anhedonia, with close to 10% of youth reporting that these 2 cardinal symptoms of depression occurred “often” or “very often.” For parent report, the most commonly endorsed symptoms were low energy and feelings of worthlessness; 14.6% of parents reported that their children experienced low energy “often” or “very often” and 6.8% of parents reported their children often felt inferior or worthless.

Correlations between measures of celiac disease and depression and youth reported quality of life are presented in Table 2. There were no associations between age, duration with celiac disease, duration on a GFD, or current gastrointestinal symptoms and quality of life. There were no statistically significant correlations between adolescent perception of celiac disease state and quality of life. There was a significant association between parental report of adolescent’s adjustment to celiac depressive symptoms and youth report of quality of life (*r* = –0.19*, P* < 0.05). A moderate association was observed between adolescent report of depression and quality of life (*r* = 0.59, *P* < 0.01) as well as parental perception of depression and quality of life (*r* = 0.41, *P* < 0.01). Linear regression models revealed that only depressive symptoms by youth and parent report, and not adjustment to celiac, explained unique variance in quality of life (see Table 3).

**TABLE 2.** Bivariate associations with quality of life

	Quality of life (youth report)
Age at time of survey	–0.04
Age at diagnosis	0.08
Years on GFD	–0.11
Depression score (youth)	0.59^†^
Depression score (parent)	0.41^†^
Adjustment to celiac (youth)	–0.15
Adjustment to celiac (parent)	–0.19*
Current Gastrointestical Symptoms	Mean (SD) Score on Quality of Life
Yes	44.0 (2.4)
No	39.8 (1.6

*P < 0.05.

†*P* < 0.01.

GFD = gluten-free diet.

**TABLE 3.** Regression models

Quality of life (youth report)			
	Standardized beta	t-score	Significance
Adjustment to celiac (parent-report)	–0.072	–0.776	0.439
Depression symptoms (parent-report)	0.418	4.49	<0.001
Overall F (df = 2, 100) = 12.18, *P* < 001, adjusted R square = 0.170			
**Quality of life (youth report**)			
	**Standardized beta**	**t-score**	**Significance**
Adjustment to celiac (youth-report)	–0.117	–1.52	0.131
Depression symptoms (youth-report)	0.611	7.95	<0.001

Overall F (df = 2, 102) = 34.46, *P* < 0.001, adjusted R square = 0.392.

## DISCUSSION

Although celiac disease primarily affects the gut, the clinical manifestations of the disease are diverse with many possible extraintestinal systems affected, including emotional functioning (10). Our study sought to further examine what factors may determine quality of life in adolescent celiac patients, ages 12–18 on a GFD with the hope of using this information to generate a future study correlating these findings with long-term adherence to the GFD in the adolescent/young adult population, known to be at high risk for diet noncompliance (4).

Despite our hypothesis that factors such as age, duration with celiac disease, or impact of CD on diet would be statistically significantly linked to lower quality of life in adolescent celiac patients, the only factor that showed a statistically significant contribution was depression, reported both by the patients and their caregivers. The depression scores in this population of CD patients were comparable to those reported from a psychiatric clinic sample of adolescents with diagnosed mental health disorders (means = 16.2 and 13.3, respectively) (8) and twice as high as scores reported in a community sample of White and Black American girls (means = 6.6 and 6.9, respectively) (9).

Rates of psychiatric disorders, including anxiety and depression, in pediatric celiac patients, are reported to range from 5% to 10% (11, 12). In a recent study by Simsek and colleagues, rates of psychiatric symptoms in newly diagnosed celiac pediatric patients aged 9 to 16 years old were compared with age and sex-matched healthy controls who had presented for routine checkups. Results from that study revealed no statistically significant differences between depression scores in newly diagnosed CD patients and controls, although there were statistically lower scores on emotional well-being within the CD patients as compared to controls. Patients on a strict GFD, however, showed significant reductions in depression scores at a follow-up (mean interval 9.61 ± 4.35 months), compared with patients noncompliant with the diet (13). There have been very mixed results, however, regarding associations between GFD adherence and depression symptoms ranging from no change in depression (14) to moderate improvements in depression (11) to complete resolution of depression (15).

The present study is cross-sectional in design, and this cannot address the question of directionality of effects of depression and CD. Psychiatric disorders that occur after the diagnosis of CD have been associated with an impaired quality of life and difficulty adapting to the chronic nature of the disease (14). In addition, psychiatric disorders that occur before the diagnosis of CD have been hypothesized to be related to disease-related cerebral hypoperfusion (16), proinflammatory cytokines (17), and low folate levels (18). Significant differences have been observed between the gut microbiota of CD patients and healthy controls (19) and between patients with depression and those without (20). In a study of infants at high risk for CD given family history, difference in gut microbiota in infancy was predictive of disease onset later in life; children who went on to develop celiac disease had lower bacterial “diversity” over time, had reduced levels of sIgA and higher levels of *Bifidobacterium breve* and *Enterococcus* species as compared to the children who did not develop celiac disease. Similar to the findings in high-risk celiac patients that go on to develop CD versus those that do not, studies have also demonstrated that depressed patients’ microbiota differs significantly from healthy controls with reduced microbiota diversity, a reduction in Firmicute families, Bifidobacterium in addition to Faecalibacterium and Tuminococcus, and higher levels of Prevotellaceae and Prevotella (20–22). We know of no studies examining the gut microbiota in CD patients with and without depression symptoms, leaving open the possibility that 1 mechanism by which high rates of depression are observed in this adolescent population is via changes in the microbiome. Future research that directly compares adolescent CD patients to other populations, including healthy peers as well as peers with other chronic health conditions, would help to generate greater specificity in potential causes of poor quality of life.

Future research is needed to assess if and how depression impacts long-term adherence to the GFD in celiac adolescents and young adults. Studies that assess history of mental health rigorously and repeatedly are needed to help discern the direction of effect between mood- and CD-related symptoms and interventions. If depression is associated with poor dietary adherence in early adulthood, it may create a strong argument for mandatory adolescent depression screening with the hopes of targeting this “at risk” population early, implementing tools to better treat their depression (potentially psychotherapy, pharmacotherapy, or interventions to restore a healthy gut microbiota such as prebiotics, probiotics, or healthy food choices), improve adherence to the GFD, reduce long-term serious sequelae from untreated celiac disease, improve their quality of life, and improve use of health care dollar spending.

Despite the significant results of the present study, there are multiple limitations. First, all pediatric study participants were derived from a single urban academic university setting; therefore, these results may not generalize to other populations. Second, depression scores were derived from questionnaires as opposed to a structured diagnostic interview of psychiatric disorders (although these rating scales have been shown to be valid instruments to identify children with psychiatric symptoms). Third, without a group of healthy children, we are unable to compare scores and patterns of associations for depression and quality of life. Finally, although our results are compelling, the cross-sectional design did not allow for testing of the direction of effect.

## CONCLUSION

Adolescents with celiac disease report high levels of depression symptoms, with scores comparable to those reported by adolescents seeking mental health services. The length of time living with celiac disease, age at diagnosis, perception of disease state, and length of time on a gluten-free diet do not appear to correlate with quality of life. In addition to the need to address the mental health needs of celiac patients, it is possible that high rates of depression that impact quality of life leads to some of the later complications observed in the adolescent/emerging adult population, including gluten-free diet noncompliance. In summary, these data suggest that early screening for depression in any adolescent with celiac disease is crucial.
